# 930. COVID-19 symptom and viral load rebound among individuals reporting nirmatrelvir/ritonavir use compared to propensity score matched individuals not taking COVID-19 treatment

**DOI:** 10.1093/ofid/ofad500.975

**Published:** 2023-11-27

**Authors:** Sarah E Smith-Jeffcoat, Jessica E Biddle, H Keipp Talbot, Vanessa Olivo, Ellen Sano, Sara H Goodman, Joshua Petrie, Karla I Ledezma, Ayla Bullock, Suchitra Rao, Alexandra Mellis, Sheroi Johnson, Hannah L Kirking, Melissa A Rolfes, Yuwei Zhu, Jonathan Schmitz, Kimberly W Hart, Steph Battan-Wraith, Lori S Merrill, Son H McLaren, Celibell Vargas, Clea Sarnquist, Prasanthi Govindaranjan, Edward Belongia, Kathleen Pryor, Karen Lutrick, Amy Yang, Quenla Haehnel, Edwin J Asturias, Natalie M Bowman, Katherine Ellingson, Huong McLean, Yvonne A Maldonado, Melissa Stockwell, Kerry Grace Morrissey, Carlos G Grijalva, Phillip P Salvatore

**Affiliations:** Centers for Disease Control and Prevention, Atlanta, Georgia; Centers for Disease Control and Prevention, Atlanta, Georgia; Vanderbilt University Medical Center, Nashville, Tennessee; Westat, Rockville, Maryland; Columbia University Irving Medical Center, New York City, New York; Stanford University School of Medicine, San Jose, California; Marshfield Clinic Research Institute, Marshfield, Wisconsin; University of Arizona College of Medicine, Tucson, Arizona; University of North Carolina, Chapel Hill, North Carolina; University of Colorado School of Medicine, Aurora, Colorado; Centers for Disease Control and Prevention, Atlanta, Georgia; Centers for Disease Control and Prevention, Atlanta, Georgia; Division of Viral Diseases, National Center for Immunization and Respiratory Diseases, CDC, Atlanta, Georgia; Centers for Disease Control and Prevention, Atlanta, Georgia; Vanderbilt University, Nashville, Tennessee; Vanderbilt University Medical Center, Nashville, Tennessee; Vanderbilt University Medical Center, Nashville, Tennessee; Westat, Rockville, Maryland; Westat, Rockville, Maryland; Columbia University Irving Medical Center, New York City, New York; Columbia University Irving Medical Center, New York City, New York; School of Medicine, Stanford University, Palo Alto, California; Stanford University School of Medicine, San Jose, California; Marshfield Clinic Research Institute, Marshfield, Wisconsin; University of Arizona College of Medicine, Tucson, Arizona; University of Arizona College of Medicine, Tucson, Arizona; University of North Carolina, Chapel Hill, North Carolina; University of North Carolina, Chapel Hill, North Carolina; University of Colorado School of Medicine, Aurora, Colorado; University of North Carolina, Chapel Hill, North Carolina; University of Arizona, Tucson, Arizona; Marshfield Clinic Research Institute, Marshfield, Wisconsin; Stanford University, Stanford, California; Columbia University Irving Medical Center, New York City, New York; Westat, Rockville, Maryland; Vanderbilt University Medical Center, Nashville, Tennessee; Centers for Disease Control and Prevention, Atlanta, Georgia

## Abstract

**Background:**

Nirmatrelvir/ritonavir (N/R) protects against severe outcomes after SARS-CoV-2 (SCV2) infection, but patients and studies have described symptom and viral rebound after treatment. Our aim was to compare symptom and viral trajectories during acute illness among individuals with COVID-19 treated with N/R compared to similar individuals who did not receive any COVID-19 treatment.

**Methods:**

This analysis included participants enrolled ≤ 6 days of index symptom onset in a US household transmission study who tested SCV2-positive, Mar. 2022–Mar. 2023. We followed participants for 10 days after enrollment, obtaining demographics, clinical history, daily symptoms (list of 15), medications, and specimens for SCV2 quantitative PCR. Symptomatic participants eligible for N/R were included (Fig. 1). We used propensity score matching to select untreated participants who were similar to N/R treated participants (Table 1). We assessed symptoms and viral load (when ≥ 2 nasal swab results were available) from N/R completion (N/R treated) or after seven days since symptom onset (untreated) to the end of follow up. We defined symptom rebound as an increase of ≥ 2 symptoms and viral load rebound as an increase of ≥ 0.5 log_10_(IU/mL) over a minimum of 5 log_10_(IU/mL). We used Wilcoxon Test to compare mean daily symptoms and viral loads and logistic regression to calculate odds of rebound.

**Figure:**
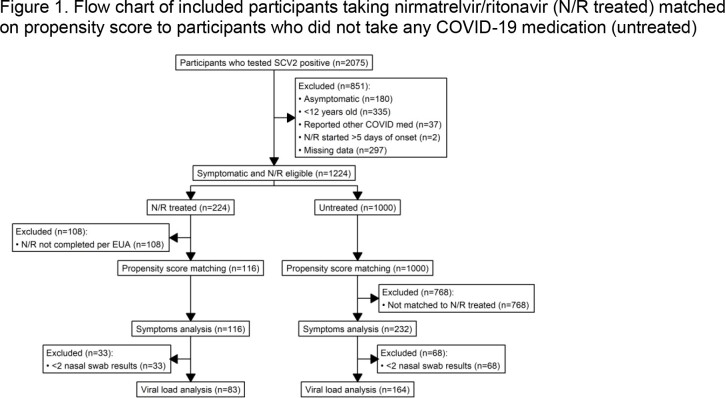

Case-ascertained household transmission study participants were included in this analysis if they were enrolled in March 2022 (first report of nirmatrelvir/ritonavir) or after and tested positive for SARS-CoV-2 (n=2075). We included symptomatic N/R eligible participants who had non-missing data for propensity score model variables and daily specimens and symptoms (n=1224) and then excluded N/R treated participants who did not complete N/R in 5-6 days according to EUA (n=108). Propensity score matching was performed by calculating propensity score of nirmatrelvir/ritonavir use based on age, sex, race/ethnicity, prior COVID-19, recruitment method, participant type, medical care access, COVID-19 vaccination history, comorbidities, and predominant variant at the time of index onset. The best covariate balance was achieved using nearest propensity score matching method with a ratio of 2:1 no treatment to N/R treated. Those that did not match to a treated participant were excluded (n=768). The two recruitment sources collected different specimen types (sentinel sites collected nasal swabs and remote recruitment collected saliva) and used different viral load quantification standards. Because of this, viral load analysis was limited to only those that collected nasal swabs and had at least two viral load results after nirmatrelvir/ritonavir completion or, for the no treatment participants, after day 7 since symptom onset. N/R=nirmatrelvir/ritonavir; SCV2=SARS-CoV-2; EUA=Emergency Use Agreement

**Figure:**
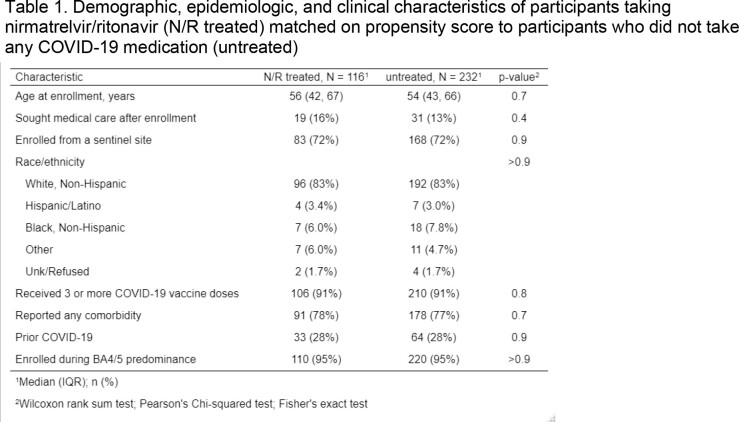

**Results:**

N/R treated (n=116) and untreated (n=232) participants had similar baseline characteristics (Table 1). Median days from symptom onset to N/R initiation was 2 days (IQR=1-3). Symptom rebound occurred among 32% of N/R treated and 19% of untreated participants (OR=1.95; 95% CI=1.17, 3.24; Fig. 2). Mean daily symptoms were lower among N/R treated (1.6 vs 2.0; p=0.2) and significantly lower among N/R treated when rebound did not occur (0.8 vs 1.5; p=0.01). Viral load rebound occurred among 25% of N/R treated and 13% of untreated participants (OR=2.31; 95% CI=1.17, 4.55) and mean daily viral load was significantly lower among N/R treated overall (1.5 vs 2.7), without rebound (1.1 vs 2.5), and with rebound (4.8 vs 5.6, all p < 0.05, Fig. 3).

**Figure:**
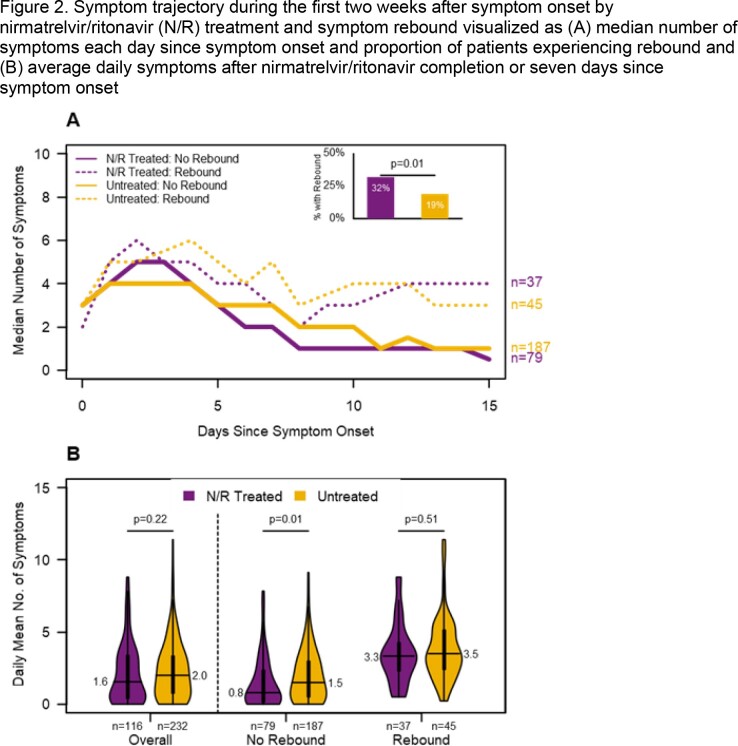

The following symptoms were elicited daily from participants: fever/feverish/chills, cough, sore throat, runny nose, nasal congestion, fatigue/feeling run down, wheezing, trouble breathing/shortness of breath, chest tightness/chest pain, loss of smell/loss of taste, headache, abdominal pain, diarrhea, vomiting, and muscle or body aches. Symptom rebound was defined as an increase of at least two symptoms after the completion of nirmatrelvir/ritonavir or, when no treatment was reported, after seven days since symptom onset. Daily symptoms after end of treatment were averaged from the day after the last day of nirmatrelvir/ritonavir or, if no treatment, day eight since symptom onset to the last available symptom diary follow up. N/R=nirmatrelvir/ritonavir

**Figure 3.**
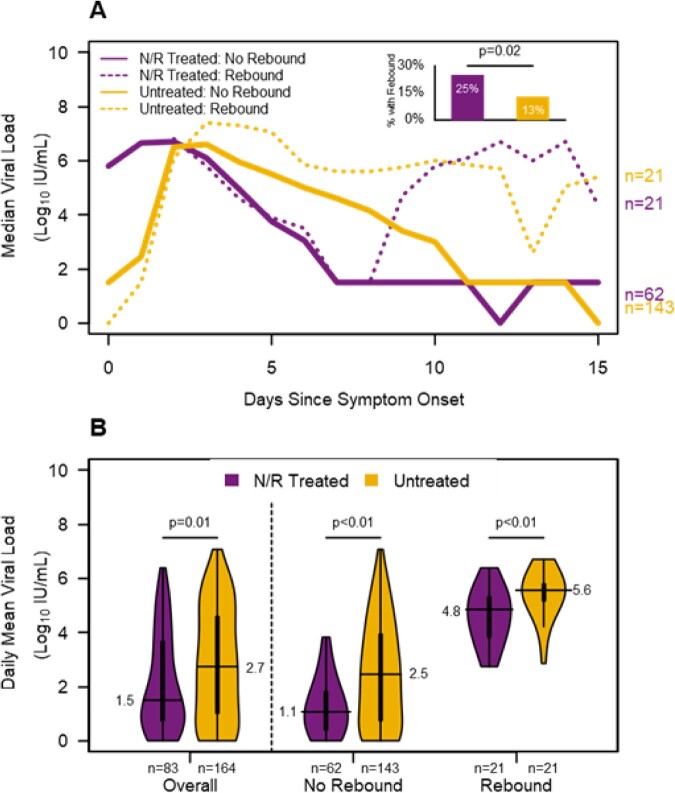
Viral load trajectory during the first two weeks after symptom onset by nirmatrelvir/ritonavir treatment and viral load rebound visualized by (A) median viral load each day since symptom onset and proportion with viral load rebound and (B) average daily viral load after nirmatrelvir/ritonavir completion or seven days since symptom onset

Nasal swabs were tested for SARS-CoV-2 by PCR using the Panther Fusion Hologic system. Viral load as logIU/mL was determined using WHO standard. Negative results were set to zero and below limit of quantification (3 logIU/mL) results were set to 1.5 logIU/mL. Viral load rebound was defined as an increase of at least 0.5 logIU/mL (with a threshold of 5 logIU/mL) after the completion of nirmatrelvir/ritonavir or, if no treatment was reported, after seven days since symptom onset. Daily viral load after end of treatment was averaged from the day after the last day of nirmatrelvir/ritonavir or, if no treatment, day eight since symptom onset to the last available viral load result. N/R=nirmatrelvir/ritonavir; IU=international units

**Conclusion:**

In outpatient settings, N/R treated individuals had fewer symptoms and lower viral loads, but greater odds of symptom and viral rebound compared to similar untreated individuals.

**Disclosures:**

**Joshua Petrie, PhD**, CSL Seqirus: Grant/Research Support **Suchitra Rao, MBBS, MSCS**, Sequiris: Advisor/Consultant **Edward Belongia, MD**, Seqirus: Grant/Research Support **Edwin J. Asturias, MD**, Hillevax: Advisor/Consultant|Moderna: Advisor/Consultant|Pfizer: Grant/Research Support **Huong McLean, PhD, MPH**, Seqirus: Grant/Research Support **Yvonne A. Maldonado, MD**, Pfizer: Grant/Research Support|Pfizer: Site Investigator, DSMB member **Carlos G. Grijalva, MD, MPH**, AHRQ: Grant/Research Support|CDC: Grant/Research Support|FDA: Grant/Research Support|Merck: Advisor/Consultant|NIH: Grant/Research Support|Syneos Health: Grant/Research Support

